# Improving health behaviours and attitudes around podoconiosis in Northern Western Ethiopia: Implementation and intervention effectiveness

**DOI:** 10.1371/journal.pntd.0012507

**Published:** 2024-09-16

**Authors:** Kibur Engdawork, Getnet Tadele, Vasso Anagnostopoulou, Papreen Nahar, Gail Davey, Shahaduz Zaman

**Affiliations:** 1 College of Social Sciences, Addis Ababa University, Addis Ababa, Ethiopia; 2 Centre for Global Health Research, Brighton and Sussex Medical School, Brighton, United Kingdom; 3 School of Public Health, Addis Ababa University, Addis Ababa, Ethiopia; Escola Bahiana de Medicina e Saúde Pública, BRAZIL

## Abstract

**Background:**

Assessing how interventions are implemented is essential to understanding why interventions may or may not achieve their intended outcomes. There is little evidence about how interventions against Neglected Tropical Diseases (NTDs) are being implemented. Guided by the Context and Implementation of Complex Intervention (CICI) framework, we evaluated an ongoing intervention against the NTD podoconiosis to examine the implementation process and its effectiveness in terms of improving shoe wearing practices, increasing knowledge and reducing stigmatizing attitudes towards podoconiosis in rural Ethiopia.

**Methods:**

We employed an exploratory mixed methods approach, qualitative followed by quantitative, between April and July 2022 to assess implementation agents, theory, strategy, process and outcomes of the intervention. We conducted document analysis, observations, focus group discussions, in-depth interviews and key informant interviews for the qualitative phase. We administered a survey to 369 rural residents, of whom 42 were affected by podoconiosis.

**Results:**

The implementers utilized government healthcare centers in a bid to mainstream podoconiosis services within local healthcare structures. The implementers provided training for health professionals and the public and distributed supplies to patients over a three-month period. The intervention reached 62% of patients, but female patients were less likely to participate than male patients. Only 18% of community members had participated in health education campaigns linked to the intervention. Involvement in the intervention resulted in improved shoe wearing practice and holding fewer stigmatizing attitudes. However, internalized stigma among patients was still rife; and the plan to utilize community assets to extend the intervention activities was not effective.

**Conclusions:**

Implementers must monitor the fidelity and progression of programs on a constant basis to make corrections. They also need to expand health education, provide psychosocial support and design economic empowerment programs for patients to reduce stigma. They must also collaborate with policy makers and international partners to sustain program activities at intervention delivery points.

## Author summary

Although many interventions against Neglected Tropical Diseases (NTDs) have been conducted in low- and middle-income countries, little is known about how intervention activities have been put into practice or their outcomes in improving health behaviour or reducing stigma. In this study, the authors examined a health intervention against one of the NTDs known as podoconiosis. The disease causes leg swelling among patients and attracts stigma. Podoconiosis can be prevented by wearing shoes consistently. The evaluated intervention was implemented by two non-governmental organizations (NGOs). The NGOs delivered the project activities using local healthcare settings and professionals. The intervention improved access to treatment and health resources for affected individuals. It also contributed to reduction of stigmatizing attitudes and improved shoe wearing practices. Despite this success, the intervention activities were not sustainable ‐ some activities were not implemented as intended and others curtailed due to limited budget. Existing socio-cultural factors such as gender inequality and low economic status of patients also created constraints. These findings may contribute to the design of complex interventions to improve the lives of people affected by NTDs.

## Introduction

Certain health conditions evoke exclusion, rejection, blame, devaluation, and other attitudes defined as stigma. These significantly impact the lives and day to day activities of those affected [1]. Among the conditions that attract public stigma are Neglected Tropical Diseases (NTDs), a group of parasitic, bacterial and chronic conditions of other aetiologies that mainly afflict individuals in poverty-stricken regions, particularly prevalent in Asia and Africa. These conditions cause devastating health, social and economic consequences among more than 1 billion people [2]. Other social determinants such as education, gender, migration and socioeconomic status increase people’s exposure to these conditions [3].

Affected individuals face health-related stigma as they are considered to have socially undesirable characteristics [4]. The physical condition of affected individuals and the fear that others have of contracting NTDs partly explain the stigma associated with NTDs. These studies have noted that stigma against patients with NTDs may exacerbate the health, social and economic impact of these conditions [5–7].

Health related stigma can be influenced by the beliefs, culture and socio-economic status of both individuals experiencing stigma and those perpetuating stigma [8,9]. Recognizing this, several social interventions have attempted to disseminate scientific evidence about the causes of NTDs to influence public discourse, facilitate contact between affected individuals and the wider public, foster positive attitudes, and empower patients economically [10–12].

One of the most stigmatized NTDs is podoconiosis, a condition that causes progressive leg swelling among affected individuals. Podoconiosis develops when genetically susceptible individuals are exposed to irritant particles in volcanic soil via walking barefoot [13]. Endemic in the highlands of Ethiopia and several other African countries, the disease is thought to affect up to 4 million people around the world and imposes physical and economic burdens on affected individuals and their family members [14,15]. Ethiopia shoulders the highest burden of podoconiosis with 1.5 million patients and 35 million people at risk [16,17]. Although podoconiosis is caused by the interaction of genetic and environmental factors [14], many rural residents believe the disease is contagious and runs in families, leading to public stigma against affected individuals. Stigma is usually manifested through exclusion of affected individuals from marriage, educational opportunities, and social participation [18–20]. The condition and the disability resulting from it are also associated with depression among patients [21].

Podoconiosis can be avoided if individuals wear shoes and wash their feet with soap and water consistently [13]. Culturally tailored strategies to enhance individuals’ understanding about the disease and their ability to take preventive actions could reduce exposure to the disease [18,22]. For those who have already developed the condition, treatment includes use of footwear, practicing foot hygiene and lymphedema management [23]. Providing health education and foot hygiene supplies reduces the consequences of the disease and increases patients’ mobility, economic productivity and quality of life [24,25].

The Ethiopian government has shown a strong commitment to tackle podoconiosis by enacting national plans [22] and developing an integrated Lymphatic Filariasis and Podoconiosis Morbidity Management and Disability Prevention Guideline [26]. Non-government organizations (NGOs) have also been implementing interventions to improve public attitudes towards affected communities and the self-esteem of affected individuals. However, little evidence exists about the implementation process or outcomes in terms of improving health behaviour and reducing stigma.

To fill this knowledge gap, we selected and evaluated an intervention being implemented in Northwestern Ethiopia by two NGOs known as International Orthodox Christian Charities (IOCC) and the National Podoconiosis Action Network (NAPAN) The intervention involved enhancing the capacity of health professionals to expand treatment services, distributing supplies to affected individuals and conducting health education to encourage preventive actions and reduce stigma over a three-month period. The study was conducted to assess the implementation dimensions of the intervention and the effectiveness of the intervention activities in improving health behaviours related to podoconiosis and reducing stigmatizing attitudes against patients.

We employed the Context and Implementation of Complex Interventions (CICI) framework to identify [27] how the implementation was implemented and its effect on understanding of podoconiosis, public stigma, shoe wearing practices and self-stigma. Our study covers five dimensions: agents, theory, strategy, process, and outcomes. The CICI framework underlines the importance of studying implementation theory to describe the causal mechanism of implementation [28] and explain the link between intervention and its outcomes [29]. The framework further posits that evaluation should analyse the implementation process, including the full execution of activities [30]. Equally important, the framework argues that implementation strategies, methods and means used by the implementers should be given attention [27]. The framework further focuses on implementation agents, i.e. implementers and targets of the intervention [27]. Exponents of the CICI framework also assert that intervention evaluation should examine implementation outcomes focusing on fidelity, acceptability and sustainability of the intervention [31].

The study also used cascade analysis to identify barriers and facilitators of the intervention. Disease control programs that demand multiple contacts with patients have used the ‘cascade’ framework to bring about desirable behavioural change, such as HIV treatment [32] and the long-term management of NCDs [33]. The cascade framework assesses progression of programmes and summarizes where intervention processes stall and impact is lost. Cascade analysis can also be used to better understand factors affecting default from health intervention [33].

## Methods

### Ethics statement

The Research Governance and Ethics Committee (RGEC) of Brighton and Sussex Medical School (BSMS) (Reference: ER/BSMS9E3G/8) and the Ethiopian Society of Sociologists, Social Workers and Anthropologists (ESSSWA) (Reference: ESSSWA 019/21) granted ethical clearance for the study. We were given permission to conduct the study by the Amhara Public Health Institute, regional and zonal health bureaus.

We used an easily understandable information sheet. Participants voluntarily took part in the study after having a clear understanding of the aims of the study. Study participants signed consent forms after agreeing to participate in the study. Participation in the study was confidential. Personal identification data were not collected by the study and participant responses were anonymized by assigning interview numbers and questionnaire ID numbers. We provided 200 ETB ($4) to each participant to compensate for their time. The data were transferred to and stored in the BSMS Social Sciences for Severe Stigmatizing Skin Diseases (the 5-S Foundation) data repository in line with the General Data Protection Regulations (GDPR).

### Study setting

The study was conducted in rural areas of Northwestern Ethiopia where a health intervention was jointly implemented by International Orthodox Christian Charities (IOCC) Ethiopia and the National Podoconiosis Action Network (NaPAN). IOCC is an NGO that has been working on podoconiosis prevention and treatment in Ethiopia since 2010. IOCC is the official humanitarian aid agency of the Assembly of Canonical Orthodox Bishops of the United States of America. Since its inception in 1992, IOCC has delivered $534 million in relief and development programs to families and communities in more than 50 countries. IOCC began working in Ethiopia in 2003 to reach remote communities and provide humanitarian assistance. As of 2010, the organization has engaged in raising awareness about podoconiosis and reducing stigma. It has also been training patients and health professionals in podoconiosis treatment [34].

Of the seven districts in which the intervention was being conducted, we purposefully selected Yilmana Densa and Dera districts, both of which were high (10%) podoconiosis prevalence districts. From these districts, we purposefully selected four *Kebeles* (smallest administrative units); namely, Shime, Agita, Fetlo and Abika *Kebeles*. About 145 affected individuals were living in these *Kebeles*.

### Study design

We employed an exploratory sequential mixed methods design. The study was conducted in two distinct phases: qualitative followed by quantitative to identify the implementation components of the interventions.

#### The qualitative phase

Documents were analysed to examine the implementation theory, strategy and agent aspect of the intervention. We analysed the “Next Steps for Podoconiosis Patients in Amhara Region, Ethiopia” project document [35]. This document contains information about the goal, objectives, strategies of the project and the outcomes it seeks to achieve.

We conducted in-depth interviews with 32 purposively affected individuals between May and June 2022 to learn about intervention targets and outcomes. After obtaining a list of affected individuals from health posts, we invited individuals within the age category of 18 to 64 years who were permanently living in the intervention area. The study involved both affected individuals who took part in the intervention and those who were not part of the intervention to compare the changes brought out by the intervention. Interviews were conducted privately in individuals’ compounds.

We observed health education programs and services at healthcare facilities. We prepared an observation guide to document how risk communication education was conducted. Two of the research team members took observation notes. Observation at healthcare centres took about 45 minutes.

We also conducted 19 key informant interviews with staff of intervention implementing NGOs, local health professionals and administrators to assess the major health issues addressed by the program, the inputs, or resources available, the methods used by the organization and by the health professionals to deliver the project activities, the process and the outputs of the intervention. Interviews were conducted in the offices of the key informants.

#### The quantitative phase

We conducted a household survey with selected household representatives. From a total of 6,439 households, we randomly selected 369 household heads, anticipating 50% variation in the outcome variables; and assuming +/- 5% accuracy and 95% confidence level. Considering 10% podoconiosis prevalence rate in the settings, we allotted 10% of the sample for affected individuals. Anticipating refusal, we oversampled the affected population and included 42 affected individuals in the survey.

Community members’ understanding of podoconiosis: Two domains of knowledge were measured: accurate understanding and non-scientific assumptions about cause and prevention action.

*Accurate Understanding*: Accurate knowledge about podoconiosis was measured by agreement with two preventive actions that can help prevent the disease (Wearing shoes on a regular basis prevents podoconiosis) and one statement that measured respondents’ belief in the preventability of podoconiosis (Children of patients of podoconiosis cannot prevent the disease: Rejecting this statement is considered to be an accurate understanding). Accurate responses were assigned one point. Accurate responses are assigned one point while inaccurate responses were assigned 0. Higher scores indicate more accurate understanding.

*Non-scientific assumptions about causes*: Non-scientific assumptions about causes of podoconiosis were measured using eight questions based on prior research conducted with individuals living in podoconiosis-endemic communities [36] and our qualitative findings. The assumptions were categorized into four subscales, a “contagion belief” based on responses to four questions assessing community members’ perception of podoconiosis as a contagious disease (e.g. “podoconiosis develops through physical contact with patients”). A second subscale was labelled “supernatural explanation” to assess whether individuals’ believed that the disease was a curse from God. The “environmental and superstitious belief” scale measured whether respondents believed that insects in the soil, stepping on goat blood or snake poison, or evil eye, caused podoconiosis, respectively. The fourth “hereditary belief” had one question that measured whether individuals believed podoconiosis to be hereditary. Each endorsement of non-scientific assumptions was assigned one score. Higher scores indicate more misconceptions.

Internalized stigma: Reported stigmatizing experience was measured using four indicators of internalized stigma (e.g., Because of your condition, you felt that your life is not satisfying; you felt that you are not useful; you felt that you don’t want to meet with other people; you felt inferior to other persons) with three scale response points. The scale showed an acceptable level of consistency (Cronbach’s alpha = 0.628).

Community members’ attitudes towards messages disiminated by the intervention: Attitude towards messages on podoconiosis was measured using seven indicators with three scale response categories (1 = Disagree; 2 = Neutral; 3 = Agree). E.g. “It is difficult to remember the major messages I have received from the training”.

Public attitudes towards patients: Public attitudes towards podoconiosis-affected individuals were measured using six indicators (e.g., “Are you willing to be a member of an *idir/mahiber* (traditional associations) with people affected by podoconiosis?”). Participants’ responses were captured using a 3-point scale (1 = Unwilling; 2 = Not sure; 3 = Willing). The indicators were taken from a prior study that adapted a social distance measure to the context of podoconiosis in Ethiopia [36]. The scale showed good consistency (Cronbach’s alpha = 0.77)

Shoe wearing practice: Reported shoe wearing practice was measured using six items that indicated shoe wearing practices in various setting and situations (at home, walking to church, market, healthcare center or towns, etc, while farming or any other workplace, during social gatherings, in the neighbourhood and in the marketplace). Responses were captured using a 3-point scale (1 = Never; 2 = Sometimes; 3 = Always). The items were found to be reliable (6 items; Cronbach’s α = 0.85).

We evaluated the effectiveness of the intervention focusing on its role in reducing misconceptions, expanding accurate understanding, improving shoe wearing practices, reducing public stigma and reducing self-stigma among patients.

We developed the questionnaire in English, translated into Amharic (the main language used in the setting), then checked for accuracy and back translated to English. Eight individuals who have first degrees in social science and related studies were recruited using informal networks, took training and administered the survey using computer tablets at respondents’ houses. The survey was completed in July 2022.

### Data analysis

The qualitative data were analysed using NVivo version 11 data analysis software. We recorded in depth and key informant interviews and then transcribed and translated into English. Observation notes were taken during each observation and expanded these into fieldnotes. Transcripts and the intervention document were imported into NVivo software and coded the data line by line considering the implementation dimensions: agents, theory, strategy, and process. We also conducted evaluation coding to compare how the programs measured up to what was planned. The codes were then thematically arranged to consolidate meaning and explanations in line with the research questions (See supplementary material, S1 Data). Tablets were used to record survey responses. We then transferred the data into the REDCap application. The data were exported to Stata version 17 and SPSS version 26 software (See supplementary material, S2 Data). We checked the data for missing values and inconsistencies and executed univariate, bivariate, and multivariate analyses on survey responses. This helped to indicate the behavioural and attitudinal changes brought about the intervention. We also conducted cascade framework analysis to learn about barriers and facilitators to reduce internalized stigma among patients.

We tried to establish the trustworthiness of our qualitative data by having a maximum variety of views on the implementation of the intervention. Data collectors visited patients at least twice at their homes. Interviews were audio-recorded, and the lead author took field notes during observation. After the completion of interviews, data collectors summarized the main points and read them back to interviewees for confirmation. The data were transcribed into the local language (*Amharic)* and translated verbatim into English and the lead author coded the data. The quantitative data were collected and analysed rigorously. To assure the reliability of the survey data, the study used previously published measures on knowledge about podoconiosis and stigmatizing attitudes, and developed indicators using the qualitative data collected in advance. Trained data collectors conducted interviews, and the lead author supervised the data collection process. Cronbach’s alpha test was conducted to measure the internal consistency of indicators. Attempts were made to assure the validity of the survey measures. The items were in consonance with some of the domains of the CICI framework. All authors evaluated the survey measures. We randomly selected participants to assure the external validity of the study.

## Results

We involved 32 affected individuals, fourteen of whom were females in the qualitative phase of the study. Most (28) individuals had no formal education and were engaged in agricultural activities A total of 369 individuals identified through random sampling techniques agreed to take part in the survey. The majority of the survey participants (77%) were males, and 11% were affected by podoconiosis. Table 1 shows that three in four did not have formal education. The mean age of the survey participant was 46 (SD = 12.6). Farming was reported to be the major occupation by 95% of the respondents. About 78% of the respondents reported that health extension workers (HEWs) had visited them at home at least once in the 6 months prior to the survey. The average number of visits to health care facilities in the three months prior to the survey was 1.7 (SD = 2.3).

**Table 1:** Socio-Demographic variables of survey participants.

Variables		N(369)	%
**Gender**	Female	86	23.3
	Male	283	76.7
**Level of education**	No formal education	277	75.0
	Grade 1–6	61	16.5
	Grades 7–8	27	7.3
	Grades 9–10	1	0.3
	Grades 11–12	3	0.8
**Affected status**	Affected	42	11.4
	Unaffected	327	88.6
**Occupation**	Farming	349	94.6
	Small business	18	4.9
	Daily labour	2	0.5
**Visited by Health Extension Workers 6 months prior to the survey**		286	77.5
**Average time to commute to healthcare facilities**	Mean minutes	76.2 (SD = 85.4)	
**Age**	Mean age	46.0 (SD = 12.6)	

### Implementation agents

#### Funders

The IZUMI Foundation was the major funder of the evaluated intervention. A key informant said, “IZUMI foundation is our longstanding funder since 2016” (KII, staff of IOCC). IOCC International was also another donor.

Gray documents show that NGOs working on podoconiosis prevention and control had been operating dispersedly until 2012. The NGOs, including IOCC, established the National Podoconiosis Action Network [37], an umbrella organization that coordinates efforts to prevent and control podoconiosis. The organization also facilitates learning forums and builds the capacity of the consortium [37]. According to a key informant, IOCC is a founding member and a strong partner of NaPAN. IOCC staff members serve as board members of NaPAN and provide technical support for NaPAN. IOCC staff also assist NaPAN in resource mobilization and related activities. The staff of the two organizations meet on a regular basis to develop intervention proposals. NaPAN and the IOCC have so far jointly implemented social interventions on podoconiosis in the Gondar Zone of Amhara region. IOCC was responsible for implementing the evaluated intervention activities whereas NaPAN supervised and evaluated the implementation of the project activities.

#### Intervention targets

The intervention targeted local health professionals, individuals affected by podoconiosis and wider local community members. The NGOs attempted to enhance local health professionals’ skill in podoconiosis disease management. They reached affected individuals with self-management and disability prevention training. The organizations also targeted the wider community to enhance prevention actions and reduce public stigma [35].

### Implementation theory

The project document revealed the NGOs intended to improve the lives of affected individuals by improving the quality of and access to care and extending the reach of services. The implementers assumed that utilizing community resources and local healthcare professionals would help expand health services to affected individuals. They further assumed that enhancing public understanding of podoconiosis would improve adoption of preventive actions such as wearing shoes and public stigma [35].

### Implementation strategy

The document analysis revealed that IOCC and NaPAN employed an integrative model; utilizing local healthcare centres in a bid to mainstream podoconiosis services into local structures. An IOCC staff said, “The project was informed by IOCC’s past intervention evaluations that suggested standalone or direct projects were less cost effective” (KII, staff of IOCC). Another key informant from the Regional Health Bureau also said, “The project employed an integrative approach with the intention to link lymphedema service with the local health structures. This is in line with the national NTD Master plan, and it serves as a learning exercise for future interventions” (KII Regional NTD team leader).

The implementers believed that the strategy minimized duplication of efforts and improved the efficiency of the project. In addition, the approach helped to develop the capacity of local health professionals and strengthened collaboration between NGOs and local healthcare providers. A key informant said, “This approach created advantages in sharing resources, experience and knowledge between NGO staff and local health professionals” (KII, staff of IOCC).

The NGOs aimed to integrate gender and disability issues into their intervention activities. The project document, for example, explains that, “The project intends to help treat vulnerable and at-risk population groups with a particular focus on marginalized female patients” [35].

### Implementation process

#### Cascade training

The two NGOs jointly conducted a three-day cascade training with 183 health professionals in September 2020 to familiarize health professionals with podoconiosis and enhance their capacity to provide treatment services to affected individuals. Most of the health professionals had positive remarks about the training as demonstrated by the following quote, “I would say that the training has opened my eyes about podoconiosis treatment. The training was supported with visual aids, video, and group exercises” (KII, Podoconiosis focal person, Yilmana Densa District).

Some of the trained health professionals in turn provided training for HEWs at healthcare facilities. A health professional stated, “After we took part in the training, we informed health extension workers about the purpose of the intervention and their specific role in the intervention” (KII, health professionals, Dera District). This claim was echoed by some of the HEWs who received training from the trained health professionals. A HEW said, “A trained focal person gave us a half-day training on podoconiosis. He gave us an orientation about the disease using training manuals” (HEWs, Yilmana Densa District).

#### Distribution of treatment supplies and provision of care and treatment

Utilizing local health professionals and health facilities, the intervention implementers distributed supplies such as soap, washing basins and shoes to affected individuals over a three-month period. The health professionals demonstrated self-care management at healthcare facilities. Of the 42 affected individuals who took part in the survey, 26 (61.9%) participated in the health intervention activities and reported that they had received health resources. Most of the participants in the qualitative interviews also reported taking part in the interventions. An affected person stated, “I went to a health station three times, as all patients in the village were invited by HEWs. I was given soap, Vaseline and a plastic washing basin. The health professionals washed my feet with soap. They advised me to wash my feet every day. I also received shoes and soap” (IDI, affected male, age 18). Another affected person noted, “The health staff soaked our feet in water in a plastic bowl and showed us how to wash our feet” (IDI, affected male, age 25).

The foot-washing demonstration sessions created an opportunity to put across a message to the community that podoconiosis is not contagious. This was reflected in the following excerpt from an FGD participant, “I have seen health professionals washing affected individuals’ feet, touching them with their hands. It gave me the lesson that it is not transmittable with skin contact” (FGD participant, male, Dera district). The intervention also created the impression among most patients and non-patients that podoconiosis was a treatable condition. A quote from an FGD participant demonstrates this claim, “In the past, we thought that this disease could not be treated. Now we have seen the disease can be treated and cured. Some patients have recovered from it” (FGD participant, female, Yilmana Densa District).

The intervention required affected individuals to attend services at healthcare centres at least three times (once in every month). On completion of these three visits, affected individuals ‘graduate’ from the intervention and are expected to continue self-care procedures at home. A few affected individuals with large nodules also received nodulectomy operations. We observed five patients receiving this treatment at healthcare facilities in Dera District. According to these people, the procedure improved their physical health and enabled them to wear shoes. An affected person stated, “After my operation, my foot condition improved, and I was able to wear shoes. I even started to take part in social activities” (IDI, affected male, age 64).

#### Establishing patient associations

The NGOs coordinated the establishment of patient associations in the two communities. According to the implementers, the aim of establishing patient associations was to create a platform where affected individuals could share experiences and create a mechanism to get social and economic support. By doing so, affected individuals can manage their physical and social wellbeing. An affected person remarked, “The aim of forming the association was to establish our own institution to help each other. We were informed by the staff that the support from the health centre may not last long” (IDI, affected female, age 56). The patient association required members to hold regular meetings every month to discuss challenges, share experiences and promote their priorities. However, most of the associations were not functional during the data collection period and affected individuals saw little relevance in them. An affected individual noted, “Before the association stood on its feet, most patients started complaining about its lack of support. Although members started contributing 5 Ethiopian Birr each month, they did not see any benefit. They demanded instant support, and some patients withdrew from membership taking their money back” (IDI, affected female, age 56).

#### Risk communication

**Fig 1:**
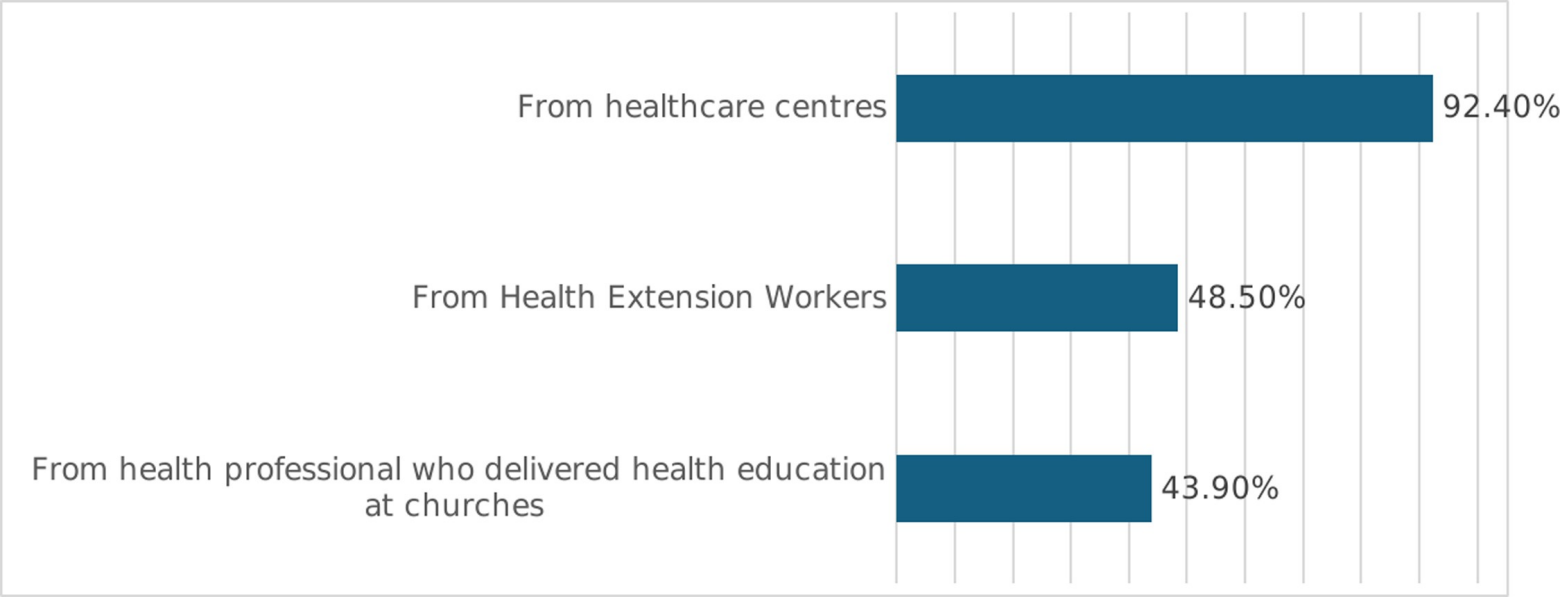
Source of information about podoconiosis for those who received it (N = 66).

Trained health professionals disseminated information about the causes and prevention of podoconiosis to community members. We observed how health professionals provided health education at healthcare facilities. On the morning of 29^th^ of May 2022, we observed a risk communication campaign in *Agita* health care centre:

A male health officer with a white gown came to the waiting area, asked the patients to pay attention and introduced them to the health topic of the day i.e., podoconiosis. He explained how walking barefoot on “acidic soil” causes the condition. In between his messages, the health officer used some jargon and English words such as gland, bacteria, podoconiosis. The health officer informed residents in the centre that some individuals are highly susceptible to irritation caused by the “acidic soil” due to genetic factors. It appeared that the point was not well explained, and participants barely captured this complex issue. The health professional also underlined to the audience that the disease could be treated if immediate action was taken at the early stage. He also stated that podoconiosis was not contagious and advised the audience to wear shoes regularly to prevent the disease. In the end, he asked if there were any questions or points needing clarifications. No one asked questions or responded to the questions asked by the officer partly because they did not comprehend his message. Finally, the health staff wound up the session by recapping the key points. The survey revealed that only 66 (17.9%) of the respondents had received information about podoconiosis from the intervention. Of these, 61 (92.4%) reported to have received information from healthcare centres while 32 (48.5%) respondents said that they have received similar information from HEWs during home visits (Fig 1).

### Implementation outcomes

#### Fidelity

The NGOs were able to train local health professionals and start the integration of podoconiosis care within existing health facilities. Members of staff of the NGOs were of the opinion that they executed all of the intervention activities in line with the initial plans. A member of the team of implementers noted, “We found that most of the health centres were doing well … Cascade training had been implemented in most of the health centres. In many of the health centres some patients show up regularly for follow up visits. High attendance means there is good linkage with the health extension workers” (KII, staff of intervention implementing organization).

However, interviews with HEWs and local health professionals suggested otherwise. Although the intervention was designed to cascade treatment activities through existing health structures, it was mainly concentrated at the healthcare settings and did not trickle down to HEWs at health posts. A key informant noted, “The intervention was not functional at the health post level. It was limited to the health centre. We did not check patients after the three rounds of intervention at the health centre” (KII, NTD Officer, Yilmana Densa district). The following quote from a HEW further strengthened this finding, “We have not received any training on podoconiosis. It was given only to the health staff at the health centre. We were given just a quick briefing by the health staff and observed the demonstrations performed by the health staff on how to wash and bandage the affected part of the feet” (KII, HEWs at Dera District).

There were some planned activities that the implementers did not execute as intended. The NGOs had planned to follow a grassroots approach such as utilizing schools to eliminate misconceptions, enhance shoe wearing practices and reduce stigmatizing attitudes. However, the plan was not put into action and health education campaigns were not conducted in schools. NaPAN and the IOCC had also planned to follow a gender transformative approach to outreach female patients and improve their lives. However, the intervention implementers did not implement any gender sensitive activities. The intervention implementers reported that limited funding created a constraint to implement all the planned activities as intended. Limiting funding also prevented the intervention implementers from conducting a follow up assessment.

#### Acceptability

Most affected individuals were aware of the intervention. On the other hand, the wider community had limited information about the intervention. As previously mentioned, 62% of affected individuals took part in the intervention activities. This was considered to be high enrolment in the intervention. A health professional remarked, “We did invite affected individuals from households, churches, and schools. Accordingly, there was high turnout out of patients at the beginning” (KII Podoconiosis focal staff, Dera district). Most of the affected individuals reported that they had continued to practice the healthcare instructions they have received from the intervention.

Table 2 shows that only 66 respondents had received health education about podoconiosis. Among these, 36 were unaffected members of the society. A significant majority of these respondents (86.4%) reported that the training messages were clear, and similarly 86.4% stated that it was easy to implement preventive actions, while 75.8% believed that the training messages were helpful to reduce stigmatizing attitudes against patients. On the other hand, less than a half (48.5%) believed that the training messages were easy to pass on to peers.

**Table 2:** The community’s attitudes towards the health education.

Statements	n and % in agreement (N = 66)
It is easy to understand the messages of the health education	57 (86.4%)
It is easy to implement what the health professionals say to prevent oneself from podoconiosis	57 (86.4%)
It is easy to remember the major messages I have received from the training	54 (84.8%)
The information from the health professionals can reduce stigmatizing attitudes against patients of podoconiosis	50 (75.8%)
The training I have been taking has reduced the misconceptions I had about podoconiosis	49 (74.2%)
The training I have been taking can help improve the relationship between affected and unaffected individuals	47 (71.2%)
It is easy to disseminate the message I have received to my peers	32 (48.5%)

Most individuals in the two communities harbored misconceptions about podoconiosis. Involvement in the intervention was not associated with having accurate understanding about preventive actions or fewer misconceptions about the causes of the disease. Pearson’s correlation test was used to examine the association between involvement in the intervention and understanding of podoconiosis. Table 3 shows that there was no evidence that involvement in the intervention was correlated with scores on superstitious beliefs (r = 0.07, *p* = 0.22), preventive action knowledge (r = 0.09, *p* = 0.09), contagious beliefs (r = -0.06, *p* = 0.30), supernatural explanations (r = -0.02, *p* = 0.72), hereditary beliefs (r = -0.08, *p* = 0.17) or environmental beliefs (r = -0.04, *p* = 0.47).

**Table 3:** Correlation between involvement in the intervention, superstitious beliefs, preventive action knowledge, contagious beliefs, hereditary beliefs and environmental beliefs.

Variables	1	2	3	4	5	6	7
1. Involvement in the intervention	-						
2. Superstitious beliefs	0.07	-					
3. Preventive action knowledge	0.09	-0.07	-				
4. Contagion beliefs	-0.06	0.21**	0.16**	-			
5. Supernatural explanations	-0.02	0.07	0.04	-0.10	-		
6. Heredity beliefs	-0.08	0.15*	-0.17**	0.34**	-0.00	-	
7. Environmental beliefs	-0.04	-0.04	0.09	0.21**	-0.05	0.12*	-
Mean	0.18	0.2	2.44	2.83	0.77	0.59	0.94
SD	0.38	0.39	0.69	1.13	0.42	0.49	0.24
Range	0–1	0–1	0–3	0–4	0–1	0–1	0–1

*. Correlation is significant at the 0.05 level (2-tailed).

**. Correlation is significant at the 0.01 level (2-tailed).

Stigmatizing attitudes and shoe wearing practices were correlated with a few variables including involvement in the evaluated intervention. Shoe wearing score and stigmatizing attitudes were checked for normality; and the Shapiro-Wilk test indicated that scores were not normally distributed across the population (stigmatizing attitudes W = 8.93 p<0.001; shoe wearing practice score W = 0.85 *p*<0.01). Thus, a non-parametric regression kernel test was conducted.

Table 4 shows that involvement in the intervention was positively associated with shoe wearing practice. Respondents who took part in the intervention had a coefficient of 1.016 (*p* = 0.001), indicating that individuals who were involved in the intervention were more likely to wear shoes. Wealth was also associated with better shoe wearing practices with a coefficient of 0.156 (*p* = 0.030).

**Table 4:** Results of Kernel regression analysis to examine the effect of age, wealth index, gender, involvement in the intervention and educational background on shoe wearing practices (N = 364).

						Percentile	
	Shoe wearing	Observed estimate	Bootstrap std. err.	Z	P>|z|	Lower	Upper
Mean	Shoe wearing	9.207	0.16	56.92	<0.001	8.861	9.516
Effect							
	Age	-0.023	0.014	-1.65	0.093	-0.050	0.005
	Wealth index	0.156	0.005	2.95	0.030	0.007	0.027
	Gender (0 = Female, 1 = Male)	0.226	0.452	0.48	0.631	-0.638	1.171
	Involvement in the intervention (0 = Not involved, 1 = Involved)	1.016	0.344	3.21	0.001	0.505	1.853
	Education (0 = No formal education, 1 = Formal education)	0.334	0.544	0.61	0.531	-0.733	1.407

In-depth interview participants reported that improved shoe wearing practices and adherence to self-care procedures enhanced their health. An affected male noted, “I have seen improvement among those who received shoes and soap. Their feet became clean and neat. Unlike the past, their feet have no bad odor, and they are not ashamed of themselves” (IDI-6, Male patient, age 40). This point was expanded by the following quote from a health professional “There is a stark difference compared to the past [before the intervention]. We have observed improvement among many patients with regards to the occurrence of acute attacks and work-related activities” (KII, NTD officer, Dera district).

We also fitted a kernel regression to examine the predictive ability of variables on stigmatizing attitudes (Table 5). Involvement in the intervention was negatively associated with stigmatizing attitudes, with a coefficient value of -1.23 (*p* = 0.031). Education was also negatively associated with stigmatizing attitudes with a coefficient value of -1.67 (*p* = 0.003).

**Table 5:** Results of Kernel regression analysis to examine the effect of wealth index, involvement in the intervention and educational background on stigmatizing attitudes (N = 327).

						Percentile	
	Stigmatizing attitudes	Observed estimate	Bootstrap std. err.	Z	P>|z|	Lower	Upper
Mean	Stigmatizing attitudes	11.79	0.199	60.58	<0.001	11.40	12.14
Effect							
	Wealth index	-0.00	0.00	-1.63	0.174	-0.019	0.001
	Involvement in the intervention (0 = Not involved, 1 = Involved)	-1.23	0.593	-2.07	0.031	-2.377	0.086
	Education (0 = No formal education, 1 = Formal education)	-1.67	0.408	-4.10	0.003	-2.527	-0.972

On the other hand, the survey showed that involvement in the intervention did not help to reduce acute attacks or internalized stigma among affected individuals. As shown in Table 6, there was no significant difference between the number of acute attacks among affected individuals who were not involved in the intervention and those who did take part *(mean rank 19*.*28 vs 22*.*87*, *U = 172*.*5*, *p = 0*.*34)*. The intervention did not reduce the pervasive internalized stigma among patients. The Mann-Whitney test indicated that there was no significant difference between internalized stigma among affected individuals who were not involved in the intervention and those who were (*mean rank 18*.*9 vs 23*, *U = 167*.*5*, *p = 0*.*28)*.

**Table 6:** Difference in mean rank values for number of acute attacks and internalized stigma scores between affected individuals who were involved in the intervention and those who were not.

Dependent variable	Group	Mean rank	Sum of rank	Mann-Whitney U	Z	P
Number of acute attacks in 3 months prior to the survey	Not involved (N = 16)	19.28	308.5	172.5	-0.94	0.34
	Involved (N = 26)	22.87	594.5			
Internalized stigma score	Not involved (N = 16)	18.97	303.5	167.5	-1	0.28
	Involved (N = 26)	23.06	599.5			

#### Sustainability

The implementers assumed that mainstreaming podoconiosis into NTD structures and health centres would increase the sustainability of self-care treatment and ultimately improve the physical, social and economic wellbeing of affected individuals. A key informant noted, “Integration was taken as a means for sustainability as it provides opportunity for creating access and open doors services for patients, which was also believed to eventually contribute to stigma reduction” (KII, NTD officer). However, the services did not continue in the two communities. Local healthcare facilities were not providing treatment or counselling services at the time of data collection. An affected female said, “I went to a healthcare station after I heard about the intervention. The health professionals told me all treatment supplies had been distributed and the intervention was over” (Affected female, age 43). The health professionals reported that the healthcare facilities lacked sufficient budget to continue lymphedema services for affected individuals.

Some patients lost trust in self-care as it takes a long time to reduce the swelling and improve physical health. The expectation of instant cure coupled with lack of financial capacity to buy treatment supplies thwarted motivation to sustain self-care practices. This point was elaborated in the following quotes from affected individuals. One male said, “I gave up as the soap and ointment ran out. I can’t afford to buy soap, let alone shoes” (Affected male, age 64). An affected female also stated, “I tried to wash my feet and bandage it for a few weeks, it was useless… I thought the disease was curable, but I have not seen any change” (IDI, Female patient, age 50).

### Implementation cascade for the intervention with barriers and facilitators

**Fig 2:**
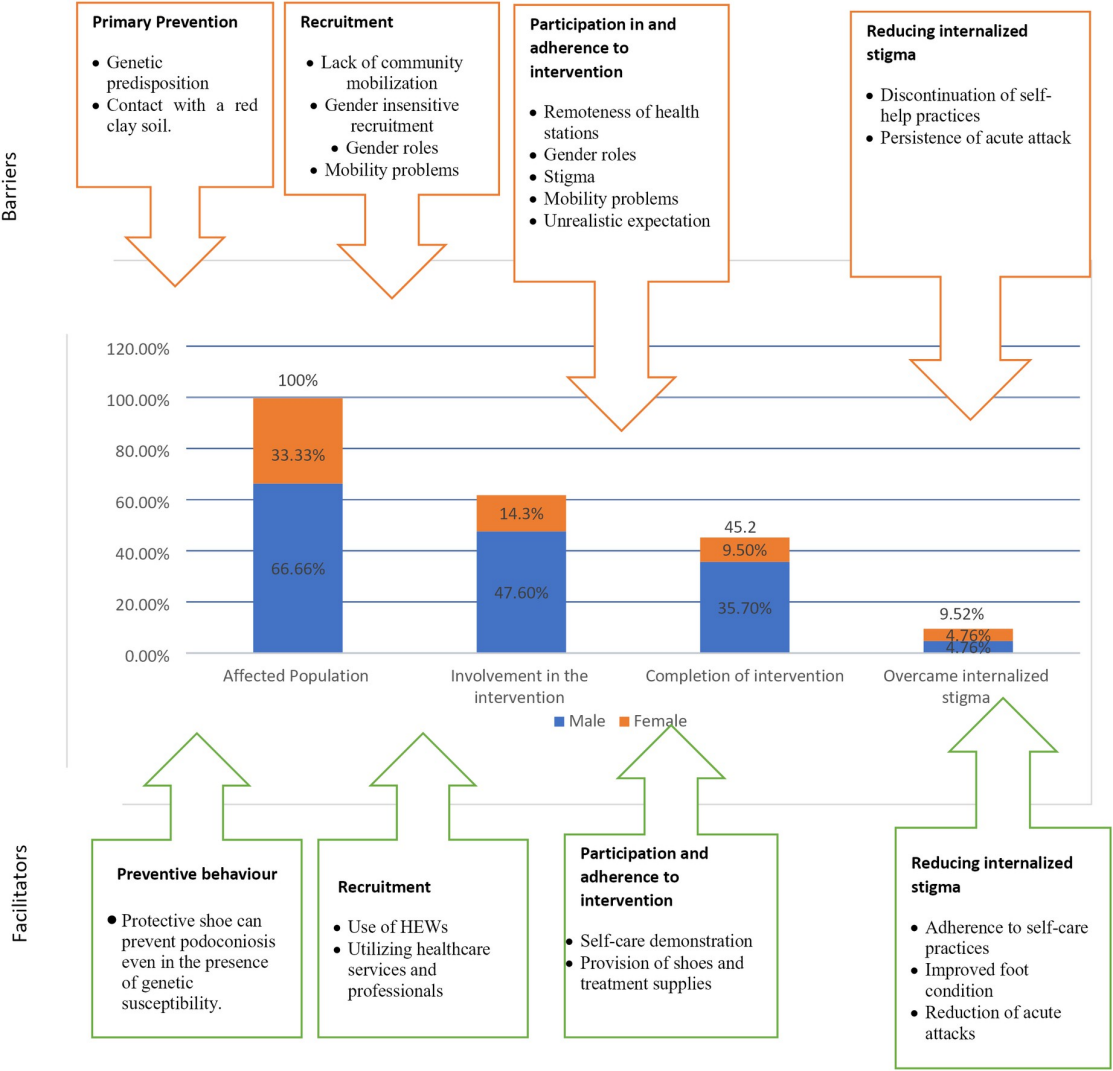
Intervention cascade with barriers and facilitators to reducing internalized stigma among individuals affected by podoconiosis.

Using the cascade framework, we outlined the major barriers and facilitators in efforts to reduce internalized stigma. The implementation cascade for the intervention is shown in Fig 2, with 14 female and 28 male affected individuals representing the 100% column calculated from the total number of affected individuals who took part in the study.

#### Primary prevention of podoconiosis

Genetic predisposition and lack of consistent shoe wearing practice have posed barriers to preventing podoconiosis. An affected female said, “My father is also affected by podoconiosis. When I was a kid, I used to walk barefoot everywhere. Shoe wearing is a recent practice. I started wearing shoes after being affected. Now I have shoes, but I often wear open plastic shoes. Closed shoes are not comfortable for work. I use closed shoes when I travel on occasion” (IDI, affected female, age 34 to 36). Since genetics is not a sole determinant of podoconiosis, reducing contact with the red clay soil could facilitate efforts to prevent the disease.

#### Recruitment and participation

HEWs went door to door and informed household heads about the intervention. Using HEWs facilitated the recruitment of affected individuals. Self-care management training was provided at healthcare facilities by local health professionals. However, as little had been done to mobilize the community, not all affected individuals joined the intervention. As the intervention activities were conducted at healthcare facilities, some affected individuals with mobility problems could not take part. Additionally, as HEWs only informed household heads about the intervention, some affected women were not aware of the intervention. An affected female reported, “My husband is also a patient. A HEW came home and told him about the intervention. He went to a healthcare station without informing me; and he received three bars of soaps and shoes. My husband was not willing to share any of the soap with me” (IDI, affected female, age 39). Affected women who did not get permission from their husbands could not also take part in the intervention activities. From a total of 42 affected individuals, 26 (61.9%) joined the intervention. Participation rate was higher among men than women: 6 females and 20 males took part in the intervention.

#### Adherence to intervention

From the total of 42 affected individuals who took part in the study, 45% completed the intervention activities: 35.7% of the men and 9.5% of the women. Health professionals demonstrated self-care practices and the intervention provided health resources such as shoes and treatment supplies. This encouraged participants to adhere to intervention activities. However, some affected individuals defaulted from the intervention due to mobility problems as they could not travel with their swollen legs. Other affected individuals reported that they faced stigma at healthcare centres and decided not to visit them. Gender also affected adherence to intervention activities. The intervention had difficulty in retaining affected females. Due to their household responsibilities, some affected females could not complete the three-monthly visits. Some affected individuals visited the healthcare facilities and took part in the intervention expecting that the intervention would instantly cure their conditions. When they realized that their condition could only be treated, some became unsatisfied with the service and defaulted from the intervention.

#### Reducing internalized stigma

Of the 42 affected individuals who took part in the study, 9.5% (half of which were female and half male) were able to reduce their internalized stigma. These individuals adhered to self-care practices thereby improving their foot condition and reducing acute attacks. Despite such success, internalized stigma remained high among affected individuals even among those who completed the intervention. This was because some individuals discontinued self-practices that might have reduced the swelling of their legs. Additionally, most of them experienced acute attacks.

## Discussion

The study described the implementation of an intervention in Ethiopia and its impact on preventive actions and stigma. Major barriers and facilitators of the intervention were identified. Assessing the implementation domain of a given intervention increases our understanding of how the intervention is put into practice and functions [27].

Employing an integrative approach, the implementers provided training for health professionals and distributed supplies to affected individuals. The training enhanced the skills of the health professionals and the health professionals provided lymphedema services for affected individuals at healthcare facilities. Trained health professionals conducted health education campaigns at healthcare facilities and churches and helped enhance community understanding about the cause and treatment of podoconiosis, improve shoe-wearing practices and reduce stigmatizing attitudes. However, the intervention could only reach a few segments of the unaffected population and female patients’ participation was low. The intervention did not reduce internalized stigma or episodes of acute attack among affected individuals. The project activities were not as effective as they could have been as services were not institutionalized into local healthcare facilities, and some affected individuals stopped practicing self-care treatment at home.

The intervention was designed and put into practice by two NGOs that have a good reputation in podoconiosis prevention and control in Ethiopia. The experience and attributes of agents exert influence on the successful implementation of the intervention, thereby affecting outcomes [27]. The reputation of the NGOs helps to get buy-in from government and other stakeholders and most of the activities were implemented in collaboration with stakeholders.

The implementers did not explicitly state their theories of change in the project document. However, as noted by Pfadenhauer et al., implementers may not always overtly state how intended changes will be brought about by their interventions [27]. Advances in implementation science have underlined the importance of using such theories to gain insights about why and how implementation becomes successful [28]. The intervention assumed that utilizing existing resources and local health professionals would help increase access to treatment, encourage adoption of healthy behaviour against podoconiosis and reduce stigmatizing attitudes. The Diffusion and Innovation Theory explains that the way in which health ideas are communicated to various community members is important in how readily they will be accepted. The implementers assumed that developing a new idea i.e., accurate understanding about the causes, prevention and treatment of the disease, would lower the odds of occurrence of unwanted events (e.g., developing the disease and stigmatizing affected individuals) [38].

Careful selection of implementation strategy plays an important role in achieving intervention goals [27]. The decision and steps taken by the NGOs to adopt their programs to utilize local health care structures to provide health services could be seen in a positive light. This may be a viable way to reach out to the affected population in a sustainable manner. Standalone projects may not influence government health services and have poor sustainability. Recent studies on skin NTDs in Africa have demonstrated that involving patients, care givers, hospital staff, outreach workers, and schools together, can provide feasible solutions to pressing health problems through shared resources within the health and social system [39–41]. On the other hand, isolated health interventions which lack linkages with established health systems are less effective in addressing health problems [42].

The implementers also established patient associations. Patient associations increased members’ understanding of treatment practices and fostered positive relations between affected individuals and unaffected community members in Southern Ethiopia [43]. Carefully planned and coordinated self-care groups of people with leprosy were also successful in encouraging members to take responsibility for wound management in Ethiopia [44]. However, the establishment of patient associations in the two communities suffered from lack of common understanding from the inception. It appears that affected individuals did not have clear ideas about the objectives of the association, leading to frustration of members and ultimately, disbanding of the associations. Implementers should carefully coordinate establishment of patient associations after building consensus on their objectives and possible outcomes. Associations may still be a viable way to create networks, increase adoption of self-care and develop opportunities for income generating activities. Implementers will need to find out the types of income generating activities that members of the self-help groups can participate in and support the development of a business plan.

Little is known about the fidelity of interventions against NTDs in LMICs. Studying how well an intervention conforms to the initial plan of delivery may help identify which components of the intervention were delivered and which were essential to a positive outcome [45]. The implementers provided most services as per their initial plan. They had planned to implement gender transformative programs and follow grassroot approaches including utilization of schools to eliminate misconceptions, enhance shoe wearing practices and reduce stigmatizing attitudes. However, these plans were not put into action. Targeting schools with health education interventions has been shown to be one of the best strategies to bring normative changes in personal hygiene among students [46]. Diminished fidelity could negatively affect the outreach and outcome of a given intervention. Experts argue that lack of implementation fidelity can negatively impact outcomes, leading to faulty conclusions about intervention effectiveness. It also can make potentially useful interventions appear ineffective [47,48]. Implementers and donors should put in place monitoring and supervision mechanisms to assure implementation of intervention programs in line with initial plans.

The assessment of intervention outcomes yielded mixed findings. While the intervention succeeded in improving shoe-wearing practices and reducing stigmatizing attitudes, it failed to enhance people’s understanding of podoconiosis. Although messages disseminated through health centres and places of worship conveyed some information to community members, the receivers noted that health professionals use difficult words. Messages that are easy, clear, interesting to understand and consistent with the values and needs of the target audience may be more easily accepted by the community [49]. The combination of over-complex messaging and existing misconceptions about podoconiosis, led to the intervention not improving the understanding of the target population. Simpler approaches can help reduce stigmatizing attitudes, as noted by a prior study in Southern Ethiopia [50].

The intervention activities were not sustained at healthcare facilities due to financial constraints. Similar constraints led individuals to not adopt self-care practices. Discontinuing care for chronic or long-term conditions is associated with an increased incidence of complications and poorer health outcomes [51,52]. Implementers must therefore aim for long term changes, which may be achieved through institutionalizing the activities within local structures [53]. This can be achieved via provision of manuals, health resources and capacity building training. Advocacy for increased political commitment for strengthening local health care facilities could also enhance the readiness of healthcare facilities to provide routine care for affected individuals.

Our study has noted that gender inequality affected participation in the intervention activities and by extension the effort to reduce internalized stigma. The intervention activities were not responsive to the needs and experiences of affected women. The gender dimensions of being affected by podoconiosis were not considered during recruitment and intervention times. In this setting, women rely on their husbands for health-seeking decisions and are usually busy performing reproductive and productive roles in their homes. The intervention activities were not tailored to accommodate these roles so women’s participation was low. The effect of gender on impact of lymphedema services in Ethiopia was also noted by Negussie *et al* [54]. Effective interventions should take the unique illness challenges of affected women into account and design a strategy that addresses these challenges. A recent study underlined the importance of addressing gender inequalities to deliver successful NTD programs at local and national levels in LMICs [55].

Addressing gaps in public knowledge is a common approach in stigma reduction [9]. The project implementers conducted health education campaigns in the communities in the hope of reducing stigma. The exclusion of patients from marriage is often associated with fear of passing the condition to children. Health education campaigns can help eliminate this pervasive misconception. However, patient stigma is not only associated with a lack of accurate understanding or fear of getting the condition from a patient. It follows that while enhancing individuals’ understanding is a very important step in stigma reduction, it may be insufficient. The physical impacts could pose a challenge to achieving good health, attractive appearance or being materially successful. These impacts can attract stigma towards patients and may not be changed by health education campaigns. Helping affected individuals to embrace their chronic condition and become successful in life could tackle the socio-cultural factors precipitating health stigma. Recent thinking [8,9,12,56] indicates that health-related stigma is a multilevel phenomenon intertwined with cultural and socioeconomic factors. Addressing structural pathways leading to stigma using a multi-level approach has shown positive results [10–12]. This study disclosed that many affected individuals suffer from internalized stigma. Internalized stigma was mainly associated with persistent acute attack and leg swelling. Adherence to self-care practices could reduce acute attack. Intervention may not always be able to completely reverse swollen legs of patients. Yet, provision of psychosocial and economic support can improve the status of affected individual and public stigma.

To bring about meaningful changes, implementers would have benefited from adopting a complex stigma-reduction strategy at intrapersonal, interpersonal and structural levels. Training on designing and implementing complex stigma-reduction could enhance the capacity of the NGOs. Beside the healthcare programs, the NGOs need to emphasize improving the lives of patients through economic and social empowerment activities and should identify community resources that can be utilized towards this end. Additionally, intervention implementers can work with existing government organizations, other NGOs working on livelihood activities and traditional institutions. The NGOs shall further aim at changing policies to alter the social determinants of podoconiosis and stigma. To raise funds for these activities, the NGOs shall continue advocating for podoconiosis control at international level and consider utilizing domestic funding.

This study identified the implementation aspect of the intervention using the CICI evaluation framework. The study could be considered as a pioneer in that it considered elements of the CICI framework in intervention evaluation against NTDs in LMICs. Employing the framework helped examine the implementation components in detail. The findings of this study can inform future implementation processes, designs, and barriers. The employment of both qualitative and quantitative methods helped reveal rich and quantifiable data about the outreach and impact of the evaluated intervention. The implementation cascade revealed the constraints and enablers of health interventions at various stages.

The study, however, had some limitations. The study focused on a single intervention, so its findings may not be generalized to other interventions with different characteristics. In addition, the study only compared the effect of participating in a health intervention between participants and non-participants within the same communities. ‘Contamination’ or spillover of elements of the intervention might make participants and non-participants appear more similar in terms of stigmatizing attitudes and preventive behaviour than they truly are. The study only focused on stigmatizing attitudes and shoe wearing practices. Future studies need to focus on the impact of health intervention on stigma and discriminatory acts and sustainable adoption of healthy behaviour.

## Conclusions

Assessing the intervention using the CICI framework enabled us to explore several dimensions of its implementation. The intervention improved access to treatment and health resources for patients, reduced stigmatizing attitudes and improved shoe wearing practices. However, the intervention activities were not sustainable ‐ some activities were not implemented as intended and others curtailed due to limited budget. To ensure the success and sustainability of podoconiosis management interventions in remote parts of rural Ethiopia, implementers need to consider a few changes. Provision of self-management items such as soap, a basin, bandages, custom made shoes and Vaseline must be considered for those under financial constraint. Home-based interventions must be offered to affected individuals with mobility problems. On top of these short-term supports, intervention implementers and the government must implement programmes which aim to improve the livelihoods of affected individuals and people at risk. Crucially for equity, gender-transformative podoconiosis management services are needed to overcome the cultural, social and economic barriers faced by women with podoconiosis in these settings. Local health professionals must consider gender roles and power during recruitment of patients and provision of services, for example making the time and place of interventions suitable for female patients. Interventions must also aim to bring a fair utilization of health resources at household levels. Health professionals must be followed by supportive supervision and refresher training, and cascade training shall be monitored. To assure the continuity of NTDs services within state healthcare systems, ongoing engagement with policy makers and international partners is vital.

## Supporting information

S1 Data Qualitative Data. (DOCX)

S2 Data (SPSS data file(.sav)). Quantitative Data. (SAV)

## References

[1] Weiss MG, Ramakrishna J, Somma D. Health-related stigma: rethinking concepts and interventions. Psychol Health Med. 2006;11(3):277–87. doi: 10.1080/13548500600595053 17130065

[2] Ending the neglect to attain the Sustainable Development Goals: A road map for neglected tropical diseases 2021–2030 [Internet]. World Health Organization 2021. Available from: https://www.who.int/publications/i/item/9789240010352.

[3] Manderson L, Aagaard-Hansen J, Allotey P, Gyapong M, Sommerfeld J. Social research on neglected diseases of poverty: continuing and emerging themes. PLoS Negl Trop Dis. 2009;3(2):e332. doi: 10.1371/journal.pntd.0000332 19238216 PMC2643480

[4] Hofstraat K, van Brakel WH. Social stigma towards neglected tropical diseases: a systematic review. Int Health. 2016;8 Suppl 1:i53–70. doi: 10.1093/inthealth/ihv071 26940310

[5] Stienstra Y, van der Graaf WT, Asamoa K, van der Werf TS. Beliefs and attitudes toward Buruli ulcer in Ghana. Am J Trop Med Hyg. 2002;67(2):207–13. doi: 10.4269/ajtmh.2002.67.207 12389949

[6] Hotez PJ. Stigma: the stealth weapon of the NTD. PLoS Negl Trop Dis. 2008;2(4):e230. doi: 10.1371/journal.pntd.0000230 18446206 PMC2322832

[7] Weiss MG. Stigma and the social burden of neglected tropical diseases. PLoS Negl Trop Dis. 2008;2(5):e237. doi: 10.1371/journal.pntd.0000237 18478049 PMC2359851

[8] Link BG, Phelan JC. Conceptualizing stigma. Annual review of Sociology. 2001;27(1):363–85.

[9] van Brakel WH, Cataldo J, Grover S, Kohrt BA, Nyblade L, Stockton M, et al. Out of the silos: identifying cross-cutting features of health-related stigma to advance measurement and intervention. BMC Med. 2019;17(1):13. doi: 10.1186/s12916-018-1245-x 30764817 PMC6376667

[10] Heijnders M, Van Der Meij S. The fight against stigma: an overview of stigma-reduction strategies and interventions. Psychol Health Med. 2006;11(3):353–63. doi: 10.1080/13548500600595327 17130071

[11] Cook JE, Purdie-Vaughns V, Meyer IH, Busch JTA. Intervening within and across levels: a multilevel approach to stigma and public health. Soc Sci Med. 2014;103:101–9. doi: 10.1016/j.socscimed.2013.09.023 24513229

[12] Rao D, Elshafei A, Nguyen M, Hatzenbuehler ML, Frey S, Go VF. A systematic review of multi-level stigma interventions: state of the science and future directions. BMC Med. 2019;17(1):41. doi: 10.1186/s12916-018-1244-y 30770756 PMC6377735

[13] Price EW, Plant DA. The significance of particle size of soils as a risk factor in the etiology of podoconiosis. Trans R Soc Trop Med Hyg. 1990;84(6):885–6. doi: 10.1016/0035-9203(90)90115-u 2096529

[14] Davey G. Podoconiosis, non-filarial elephantiasis, and lymphology. Lymphology. 2010;43(4):168–77. 21446572

[15] Davey G, Tekola F, Newport MJ. Podoconiosis: non-infectious geochemical elephantiasis. Trans R Soc Trop Med Hyg. 2007;101(12):1175–80. doi: 10.1016/j.trstmh.2007.08.013 17976670

[16] Deribe K, Cano J, Newport MJ, Golding N, Pullan RL, Sime H, et al. Mapping and Modelling the Geographical Distribution and Environmental Limits of Podoconiosis in Ethiopia. PLoS Negl Trop Dis. 2015;9(7):e0003946. doi: 10.1371/journal.pntd.0003946 26222887 PMC4519246

[17] Deribe K, Negussu N, Newport MJ, Davey G, Turner HC. The health and economic burden of podoconiosis in Ethiopia. Trans R Soc Trop Med Hyg. 2020;114(4):284–92. doi: 10.1093/trstmh/traa003 32055853 PMC7139123

[18] Ayode D, McBride CM, de Heer H, Watanabe E, Gebreyesus T, Tadele G, et al. The association of beliefs about heredity with preventive and interpersonal behaviors in communities affected by podoconiosis in rural Ethiopia. Am J Trop Med Hyg. 2012;87(4):623–30. doi: 10.4269/ajtmh.2012.12-0204 22826482 PMC3516310

[19] Molla YB, Tomczyk S, Amberbir T, Tamiru A, Davey G. Patients’ perceptions of podoconiosis causes, prevention and consequences in East and West Gojam, Northern Ethiopia. BMC Public Health. 2012;12:828. doi: 10.1186/1471-2458-12-828 23020758 PMC3519620

[20] Tora A, Franklin H, Deribe K, Reda AA, Davey G. Extent of podoconiosis-related stigma in Wolaita Zone, Southern Ethiopia: a cross-sectional study. Springerplus. 2014;3:647. doi: 10.1186/2193-1801-3-647 25485190 PMC4233027

[21] Ali O, Deribe K, Semrau M, Mengiste A, Kinfe M, Tesfaye A, et al. A cross-sectional study to evaluate depression and quality of life among patients with lymphoedema due to podoconiosis, lymphatic filariasis and leprosy. Trans R Soc Trop Med Hyg. 2020;114(12):983–94. doi: 10.1093/trstmh/traa130 33190154 PMC7738660

[22] FMoH. The third national neglected tropical diseases strategic plan 2021–2025. 2021.

[23] Podoconiosis (non-filarial lymphoedema) [Internet]. World Health Organization 2023. Available from: https://www.who.int/news-room/fact-sheets/detail/podoconiosis-(non-filarial-lymphoedema).

[24] Hounsome N, Kassahun MM, Ngari M, Berkley JA, Kivaya E, Njuguna P, et al. Cost-effectiveness and social outcomes of a community-based treatment for podoconiosis lymphoedema in the East Gojjam zone, Ethiopia. PLoS Negl Trop Dis. 2019;13(10):e0007780. doi: 10.1371/journal.pntd.0007780 31644556 PMC6808421

[25] Dellar R, Ali O, Kinfe M, Mengiste A, Davey G, Bremner S, et al. Effect of a Community-Based Holistic Care Package on Physical and Psychosocial Outcomes in People with Lower Limb Disorder Caused by Lymphatic Filariasis, Podoconiosis, and Leprosy in Ethiopia: Results from the EnDPoINT Pilot Cohort Study. Am J Trop Med Hyg. 2022;107(3):624–31. doi: 10.4269/ajtmh.21-1180 35895351 PMC9490655

[26] FMoH. Lymphatic filariasis and podoconiosis morbidity management and disability prevention guidelines. Ethiopian Ministry of Health 2016.

[27] Pfadenhauer LM, Gerhardus A, Mozygemba K, Lysdahl KB, Booth A, Hofmann B, et al. Making sense of complexity in context and implementation: the Context and Implementation of Complex Interventions (CICI) framework. Implement Sci. 2017;12(1):21. doi: 10.1186/s13012-017-0552-5 28202031 PMC5312531

[28] Nilsen P. Making sense of implementation theories, models and frameworks. Implement Sci. 2015;10:53. doi: 10.1186/s13012-015-0242-0 25895742 PMC4406164

[29] Metz A, Bartley L. Active Implementation Frameworks for Program Success: How to Use Implementation Science to Improve Outcomes for Children. Zero to Three. 2012;32:11–8.

[30] May CR, Mair F, Finch T, MacFarlane A, Dowrick C, Treweek S, et al. Development of a theory of implementation and integration: Normalization Process Theory. Implement Sci. 2009;4:29. doi: 10.1186/1748-5908-4-29 19460163 PMC2693517

[31] Gearing RE, El-Bassel N, Ghesquiere A, Baldwin S, Gillies J, Ngeow E. Major ingredients of fidelity: a review and scientific guide to improving quality of intervention research implementation. Clin Psychol Rev. 2011;31(1):79–88. doi: 10.1016/j.cpr.2010.09.007 21130938

[32] Levi J, Raymond A, Pozniak A, Vernazza P, Kohler P, Hill A. Can the UNAIDS 90-90-90 target be achieved? A systematic analysis of national HIV treatment cascades. BMJ Glob Health. 2016;1(2):e000010. doi: 10.1136/bmjgh-2015-000010 28588933 PMC5321333

[33] Fraser-Hurt N, Naseri LT, Thomsen R, Matalavea A, Ieremia-Faasili V, Reupena MS, et al. Improving services for chronic non-communicable diseases in Samoa: an implementation research study using the care cascade framework. Aust N Z J Public Health. 2022;46(1):36–45. doi: 10.1111/1753-6405.13113 34309937

[34] IOCC. Learn about us: International Orthodox Christian Charities n.d. [Available from: https://iocc.org/about.

[35] NaPAN. Next Steps for Podoconiosis Patients in Amhara Region, Ethiopia. 2020.

[36] McBride CM, Price CS, Ayode D, Tora A, Farrell D, Davey G. A Cluster Randomized Intervention Trial to Promote Shoe Use by Children at High Risk for Podoconiosis. International Journal of Health Sciences and Research. 2015;5:518–28.

[37] NaPAN. NaPAN and Podoconiosis Programs in Ethiopia. nd.

[38] Rogers EM. Diffusion of Innovations, 5th Edition: Free Press; 2003.

[39] Abass KM, van der Werf TS, Phillips RO, Sarfo FS, Abotsi J, Mireku SO, et al. Buruli ulcer control in a highly endemic district in Ghana: role of community-based surveillance volunteers. Am J Trop Med Hyg. 2015;92(1):115–7. doi: 10.4269/ajtmh.14-0405 25331802 PMC4347364

[40] Koffi AP, Yao TAK, Barogui YT, Diez G, Djakeaux S, Zahiri MH, et al. Integrated approach in the control and management of skin neglected tropical diseases in three health districts of Côte d’Ivoire. BMC Public Health. 2020;20(1):517. 32303204 10.1186/s12889-020-08632-6 PMC7164353

[41] Barogui YT, Diez G, Anagonou E, Johnson RC, Gomido IC, Amoukpo H, et al. Integrated approach in the control and management of skin neglected tropical diseases in Lalo, Benin. PLoS Negl Trop Dis. 2018;12(6):e0006584. doi: 10.1371/journal.pntd.0006584 29939988 PMC6034899

[42] Jackson SF, Perkins F, Khandor E, Cordwell L, Hamann S, Buasai S. Integrated health promotion strategies: a contribution to tackling current and future health challenges. Health Promot Int. 2006;21 Suppl 1:75–83. doi: 10.1093/heapro/dal054 17307960

[43] Davey G, Burridge E. Community-based control of a neglected tropical disease: the mossy foot treatment and prevention association. PLoS Negl Trop Dis. 2009;3(5):e424. doi: 10.1371/journal.pntd.0000424 19479039 PMC2682702

[44] Benbow C, Tamiru T. The experience of self-care groups with people affected by leprosy: ALERT, Ethiopia. Lepr Rev. 2001;72(3):311–21. doi: 10.5935/0305-7518.20010038 11715277

[45] Craig P, Dieppe P, Macintyre S, Michie S, Nazareth I, Petticrew M. Developing and evaluating complex interventions: the new Medical Research Council guidance. Bmj. 2008;337:a1655. doi: 10.1136/bmj.a1655 18824488 PMC2769032

[46] Appleby LJ, Tadesse G, Wuletawu Y, Dejene NG, Grimes JET, French MD, et al. Integrated delivery of school health interventions through the school platform: Investing for the future. PLoS Negl Trop Dis. 2019;13(1):e0006449. doi: 10.1371/journal.pntd.0006449 30703087 PMC6354954

[47] Dobson KS, Singer AR. Definitional and Practical Issues in the Assessment of Treatment Integrity. Clinical Psychology-science and Practice. 2006;12:384–7.

[48] Sánchez V, Steckler A, Nitirat P, Hallfors D, Cho H, Brodish P. Fidelity of implementation in a treatment effectiveness trial of Reconnecting Youth. Health Educ Res. 2007;22(1):95–107. doi: 10.1093/her/cyl052 16807378

[49] Morgan MG. Risk Communication: A Mental Models Approach: Cambridge University Press; 2002.

[50] Engdawork K, Davey G, Ayode D, McBride CM, Tadele G. A cross-sectional survey to assess the risk factors associated with stigmatizing attitudes towards patients with podoconiosis among rural youth in southern Ethiopia. Trans R Soc Trop Med Hyg. 2020;114(12):995–1002. doi: 10.1093/trstmh/traa091 33169168 PMC7738651

[51] Aikens JE, Piette JD. Longitudinal association between medication adherence and glycaemic control in Type 2 diabetes. Diabet Med. 2013;30(3):338–44. 23075262 10.1111/dme.12046 PMC3567301

[52] Walsh CA, Cahir C, Tecklenborg S, Byrne C, Culbertson MA, Bennett KE. The association between medication non-adherence and adverse health outcomes in ageing populations: A systematic review and meta-analysis. Br J Clin Pharmacol. 2019;85(11):2464–78. doi: 10.1111/bcp.14075 31486099 PMC6848955

[53] Shediac-Rizkallah MC, Bone LR. Planning for the sustainability of community-based health programs: conceptual frameworks and future directions for research, practice and policy. Health Educ Res. 1998;13(1):87–108. doi: 10.1093/her/13.1.87 10178339

[54] Negussie H, Molla M, Ngari M, Berkley JA, Kivaya E, Njuguna P, et al. Lymphoedema management to prevent acute dermatolymphangioadenitis in podoconiosis in northern Ethiopia (GoLBeT): a pragmatic randomised controlled trial. Lancet Glob Health. 2018;6(7):e795–e803. doi: 10.1016/S2214-109X(18)30124-4 29773516 PMC6562300

[55] Ochola EA, Karanja DMS, Elliott SJ. Local tips, global impact: community-driven measures as avenues of promoting inclusion in the control of neglected tropical diseases: a case study in Kenya. Infect Dis Poverty. 2022;11(1):88. doi: 10.1186/s40249-022-01011-w 35932055 PMC9356398

[56] Stangl AL, Earnshaw VA, Logie CH, van Brakel W, L CS, Barré I, Dovidio JF. The Health Stigma and Discrimination Framework: a global, crosscutting framework to inform research, intervention development, and policy on health-related stigmas. BMC Med. 2019;17(1):31. doi: 10.1186/s12916-019-1271-3 30764826 PMC6376797

