# Supplementary material for: Improving health behaviours and attitudes around podoconiosis in Northern Western Ethiopia: Implementation and intervention effectiveness
Source: PLoS Negl Trop Dis. 2024 Sep 16;18(9):e0012507. doi: 10.1371/journal.pntd.0012507 (PMC11426432; doi:10.1371/journal.pntd.0012507)
Supplement: S1 Data — (DOCX) [file pntd.0012507.s001.docx]

# **S1. Qualitative data**

**Improving Health Behaviours and Attitudes around Podoconiosis in Northern Western Ethiopia: Implementation and Intervention Effectiveness**

Kibur Engdawork^^[[1]](#footnote-1)^,^,Getnet Tadele^1^, Vasso Anagnostopoulou^2^ , Papreen Nahar ^2^, Gail Davey^^[[2]](#footnote-2)^,3^, Shahaduz Zaman^2^

^1^College of Social Sciences, Addis Ababa University, ^2^Centre for Global Health Research, Brighton and Sussex Medical School, ^3^School of Public Health, Addis Ababa University

Thematic text extract selection on implementation

| **Context and Implementation of Complex Intervention (CICI) dimensions**  **(All definitions were taken from the CICI framework)** | **Construct** | **Theme** | **Key findings** | **Supporting quotes** |
| --- | --- | --- | --- | --- |
| Implementation is an actively planned and deliberately initiated effort with the intention to bring a given intervention into policy and practice within a particular setting. | **Implementation goal** | Improving quality of lives. | The aim of the project was to ensure and strengthen integration of the podo operation in the target health centres. | The aim of the project is to improve the lives of individuals affected by podoconiosis in severely and heavily affected districts in the Amhara region of Ethiopia (Project document). |
|  | **Assumptions** | Mobilizing the community resources | The project activities can be successfully implemented by utilizing the community resources and actors. | - HEWs know the community even at the household level and hence they can easily identify podoconiosis cases to refer to health centers and provide patient follow-up. - Patient-led groups are living witnesses to the change they have seen as a result of the treatment they have received and hence they can easily influence other patients. - The patient-led groups/association structure could be considered as very sustainable and can be capitalized on for addressing and positively impacting podoconiosis patients and others in endemic communities. - Community leaders can access the community easily in their day-to-day rituals; and - Credible and carefully designed messages or advice they are passing onto the community are easily accepted (Project document pp 21) |
|  | **Implementation Strategy**  The implementation strategies comprise the methods (e.g. preferred and tailored activities,) and means used by the intervention implementing organization to ensure the adoption and sustainment of intervention | Integrated Approach | The NGOs utilized local healthcare centers in a bid to mainstream podoconiosis services into local structure. e. In line with IOCC global, strategic plan, IOCC Ethiopia Made a paradigm shift from direct intervention to integration model. Contextualizing it with the National NTD master plan, the main assumption was to optimize treatment impacts among podo patients through building capacity of the local health structures. | “…this approach created advantages in sharing resources and experience, minimizing resource duplications and sharing knowledges among the NTD team. The project was informed by IOCC’s past intervention evaluations that suggested standalone or direct projects were less cost effective (KII, staff of intervention implementing organization)  “Implementation design of this project employed integration approach along the line with the national Master plan with the intention to link the services with the local health structures …it serves as a learning exercise for future intervention. (KII Regional NTD team leader)  The project intends to help treat vulnerable and at-risk population groups with a particular focus on marginalized female patients (Intervention project document. |
|  | **Implementation Process**  This refers to the intervention process operationalization in the intervention implementing organization. This includes the planning of the implementation process, initial and full execution of activities, corrections, refinements and expansion of the intervention. | Cascaded training and health staff capacity building development | The intervention has familiarized health staff in local health structures with podoconiosis and enhanced their confidence to provide treatment and counseling to patients affected by podoconiosis. Some of the trained health professional provided trainings for health extension workers. | - I took part in the training conducted by the NGOs. The main topic covered all NTDs that are common in Ethiopia, but the focus was on podoconiosis, Lymphatic Filariasis and Hydrocele. The training was provided last year in 2021. Three staff form this health center, including me have been trained. I would say that the training has opened my eyes about podoconiosis. I had no information about this disease before the training. I have learned a lot from the training; gained better understanding about the causes and treatment. We have also been trained on how to provide counseling to patients. Trainers shared their rich experience on podoconiosis, and presentation were supported with visual aids, video, and group exercises (KII, Podo focal person Yilmana Densa District). - We have explained to the HEWs about the purpose of the intervention and its procedures and their specific role in the intervention. Their role was to mobilize and send patients to the health center. That was their only duty. - A trained focal person from Agita Health Center given us a half-day training on podoconiosis. He gave us orientation about the disease using training manuals (Health Extension Worker, Yilmana Densa District). |
|  |  | Distribution of treatment supplies |  | - Patients were provided with shoes, soap and washing tubs for treatment. I have seen in my eyes that patients showing up at the health center and receiving these treatments. However, I am not sure how many of them have received the support and how long the service has continued. There was such an initiative in our community (FGD with Women, Dera District). - Yes I have been enrolled. Health extension workers summoned all patients in the villages and informed us to go to the health center to take treatment. Accordingly, I went there three times, ones in every month last year. There were so many patients with me. On the first and second visit I was given soap and Vaseline oil and a plastic washing basin, and they washed my feet with soap. They informed us to wash our feet every day at home. On the third visit, I was provided with shoes and soap (IDI, affected male, age 18). |
|  |  | Treatment demonstrations | Along with introducing self-care and treatment procedure to patients, the feet washing demonstration sessions created opportunity to put across message to patients and to the community that the disease is not contagious. | - “I have seen in my eyes… Health staff washed their feet, touching them with their hands. It gave me the lesson that it is not transmittable with skin contact.” (FGD participant, male group, Dera Woreda). - The health staff were washing our feet and we were informed that when the service terminates, we should continue applying the treatment procedures at home (IDI, affected male, age 60). - During the intervention, the health staff informed us that this disease can be treatable by washing feet and wearing shoes. The health staff soaked our feet in water in a plastic bowl and showed us how to wash the feet (IDI, a 25 years old affected male) |
|  |  | Establishing Patient Association | Patients’ association have been established by in each district | The aim of the association was to establish our own institution to assist patients with economic and social support in the future. We were informed by the staff that the support from the health center may not last long (IDI, A56 years old affected female) |
|  |  | Risk communication (although intermittent) delivered at the health centers and religious settings. | Although minimal, messages disseminated at the health centers and spiritual places sparked the idea among some community members about the main cause and shoes wearing and foot hygiene as key prevention. Informants suggested extensive community level awareness to change attitude against patients. | - “Trained staff and health extension workers provided health education to the community about podoconiosis at the health center and in the churches.” (KII-06, NTD officer, Dera Woreda) |
|  | **Gaps and challenges in implementation strategy and assumptions** | Defaulting | A number of affected individuals have defaulted from the intervention activities | - They expect patients to come to healthcare centers to get the service. I couldn’t come to the health center because my village is about 3 hours far from the health center and can’t not come to the health center regularly for the service that was not helpful and effective ((IDI, a 25 years old affected male) - I went to the health station as we were told by the HEWs a new medicine has arrived. The health professionals showed us how to take care of our feet and provide us with soaps. I didn’t go in the second round. Why would I travel with my ill leg for three bars of soap? (A 45 years old affected man) |
|  |  | Staff turnover | Staff turnover at healthcare center. Trained health professionals quitted and that left a gap in implementing Lymphedema services. | - Staff turnover was another main challenge faced in the implementation process (KII, Intervention implementing organization) |
|  |  | Lack of community mobilization | There has been little attempt to mobilize the wider community in the project. | - The other component lacking in this project was a robust community awareness . A one time shot many not be helpful to bring sustainable change. A well-planned community campaigns involving religious and community leaders might be crucial to the achieve community-wide adherence to treatments and prevention practices (KII, staff of intervention implementing organization). - Community participation was low. It would have been better if we could strongly work with the local influential people in the community to change attitude of the people about shoes wearing and social stigma against patients. continuous awareness creation and training should be provided to the community leaders and volunteers in the community to bring a better change. The nature of this disease requires a lengthy interventions period of treatment, and it takes time so, continuous awareness should be given to patients and encourage role models who made changes through such interventions to share experiences to other patients( KII, NTD Officer at Dera District). |
|  |  | Lack of follow ups | The health staff didn’t make a sufficient attempt to follow adoption of treatment and other activities | - Podoconiosis intervention requires continuous effort as it take long time to cure the disease. I cannot say that our intervention for three months alone brought significant impact. I am telling you just from my observation. Formal evaluation has not been done. Even at the health center and woreda level, podoconiosis has been ignored in the regular monitoring and follow up unlike other health services (KII, podo focat person, Yilmana Densa District) |
|  |  | Overburdened and unmotivated staff | The staff at intervention delivery points were overburdened by other activities and were less motivated to implement services | - Not only the staff but also the woreda health office has given little attention towards this intervention. It was just like a onetime shot, and no one was concerned about the follow up and continuation of the intervention. Staff seem to be faded up and relinquished their responsibility. The other challenge was workload that I have in my office. I have multiple roles to play in this health center. For instance, I’m the coordinator of the health extension program, I also coordinate the youth reproductive health program, and participate in recurrent national and regional health campaigns and vaccination programs alongside with my regular duty as clinical nurse treating outpatients every day. Podoconiosis is additional load to my job although I am highly concerned about the problem (KII, Podo focal person at Yilman Densa District). - Health professional sometime were overburdened with recurrent health promotion campaigns in other health programs (KII, Staff of Intervention Implementing Organization). - During the review meeting with health centers, all the centers have reported that they have done the cascading, but with some challenges specially complaints from health extension workers for incentives have been made. HEWs are given incentive when they administered drugs. There is no incentive for them to work on podo. As a result, they are less motivated to focus on podo (KII, Staff of Intervention implementing organization) |
|  |  | Some patients felt that the intervention can not meet their needs |  | - In my opinion, this treatment it is not sufficient. I wish if higher lever government officials could have visited us to find the lasting solution for this problem. This treatment only helped to minimize the swelling. It does not help patients who have hereditary predispositions like in our family. The health center staff provided us advice only washing feet could make a difference. I don’t think this is true. They do not provide us medicines to treat the disease. I don’t think washing feet alone is the best treatment. We need a better medication to cure the disease. Washing the feet and taking care of the feet with soap and massaging it with oil requires frequent and consistent attention every day (IDI, a father of a female patient, age 63). - Frankly speaking, the benefit was not that much promising. There is only a slight change on the swelling. Of course, my feet have become smooth and free of wounds but the swelling still bigger and sometimes I experience acute attacks (IDI, a 18 years old affected male). - Patients often come to the center with the expectation to receive material support and disregarding the education session and treatment procedures. |
|  |  | Non-functionality of local structures | The local structures that the NGOs had planned to utilize to execute the project activities were not functional. | I have doubt that Health Development Army leaders may have not been reached through this process. I have also a doubt that HDAs and the structure itself was not as active as it should be. Had they been well trained, they could have mobilized more patients and facilitated follow ups at the household level. These is one of the major gaps we realized form our supervision reports. |
|  |  | Budget constraints | Budget constraints had affected the outreach and execution of the intervention | - Budget shortage for the NTD section. Although the structure is there at zonal and district level budget has not been earmarked to this structure. The project itself has been implemented with limited amount of budget. Our plan was to reach 7000 patients. However due to limited resource we were not able to reach all the patients. Specially shoe distribution was affected by the shortage of budgets. My main concern is the resource constraint. After April 2023 we will hand over the project to the health Bureau. Unless the bureau allocates budget to this initiative the continuity of the project will face the risk of termination. I would hope that the regional health bureau will take it seriously as per our memorandum of understanding (KII, Staff of intervention Implementing organization). - Based on that we have made supervisions and observed that podo focal staff in most of the health centers were actively engaged in providing the training to HEWs. We practically observed this in some health centers. In fact, we were not able to see the situation in each patient household due to budget limitations. We realized that there were variations in achievement among health centers (KII, staff of intervention implementing organization). - IOCC had planned to cover 20% of the cost for shoes and washing basin to be delivered to the poorest of the poor patients and the remaining will be contributed by the health center. (KII, staff of NGO). |
|  |  | Dependency syndrome | Free handout and inconsistent distribution of shoes and supplies created dependency and complaints among patients. | - It would have been good if all the patients have been given appropriate shoes and medical support to help them get instant cure (An affected male, age 25). - I know that some patients went to the health center, and they have received leather-made shoes washing basin and soap for treatment. Although I lately went to the center this year and asked the health staff to register me for support, they informed me that it was too late, and no service was provided at that time. I was wondering why they did not call me for such important service. I suspect health staff at the health center might have abused the budget allocated by the government for our support and refused to inform us (An affected female, age 38). |
|  |  | Non-functional patient association. | The patient association were barely functional and affected individuals see little relevance in them. | - Before the association stood on its feet, most patients started complaining about its lack of support. Although members contributed 5 Ethiopian Birr (currency) each month, they don’t see any benefit. They demanded instant support and some patients withdrew from membership taking their money back. This situation entailed discouragement among members, and I would say that currently this association is almost not existent, not functional. No one is attending the monthly meeting. Even the staff were not committed to push it consistently (IDI, affected woman, age 56) - We were informed about the importance of establish patient-led associations by IOCC. We coordinated the establishment of the association in our district. The idea was to build the economic capacity of the patients, so that hey would become self-sustained in treatment and economic life. The association didn’t progress as we expected. Members contribute 10 Ethiopian Birr per monthly. There was a high expectation of patients for a quick return from the savings leading frustrations among members and lack of trust on the association. I suspect that the staff at the *Arb gebeya* health center might have provided them an over ambitious pan of the association which didn’t live up to the patients’ expectation (KII, NTD officer Dera) |
|  |  | COVID-19 | The COVID-19 pandemic has affected the implementation of the intervention activities | - COVID-19 has affected the progress of our implementation. The outbreak of the pandemic was announced soon after we started the project. the physical distancing and containments strategies have affected mobility of the staff and all essential services at the health centers were challenged. Therefore, the joint monitoring and review meetings were not conducted due to these circumstances (KII, staff of regional health bureau). - COVID-19 was one of the major challenges we faced. We managed to provide the training for 183 trainees underneath the COVID-19 pandemic challenges where public gathering was restricted. We did this limiting the number of trainees at a time and splitting them into a small group and deploying more trainers (KII, staff of Intervention Implementing Organization) |
|  |  | Fear of Stigma |  | - I went to the health station first and I had little reason to go there for the second time….I would rather stay at home. People will stare at your feet and make you feel uncomfortable…What is the point of going to the health station? If you stay at your home, no one hurts your feeling (a 32 years old affected woman). - Most patients refrain form coming to the health centre for other health services, simply because they were ashamed of their condition. Only a few patients used to come at the health center. They don’t want to come just to avoid the reaction of people towards their feet and the bad odour that keeps people in distance from them (KII, podo focal person at Yilman Densa District). - Patients don’t want to show up on meetings organized to educate the community about podoconiosis for fear of stigma. We would rather call a separate meeting for patients in the event when we want to educate them about it or go for a home-based education. Even when we provide education in the religious institutions mixed with healthy individuals, they become the focus of attention of others and that create humiliation among patients (KII, HEWs at Yilmana Densa District) |
|  | **Implementation Agents**  Implementation agents comprise all individuals and organizations engaged with (i) deciding to implement a given intervention (e.g., funders, administrators), (ii) implementing the intervention (e.g., providers,), (iii) being the target or otherwise affected by an intervention (individuals affected by podoconiosis). | Funders | Project funder/donor agency – IZUMI Foundation  The Amhara Regional Government signatories (Health Burau, Economic planning and Finance, Bureau, Social Afairs Bureau and Education Bureau), IOCC head quarter. | “IZUMI foundation our longstanding funder to IOCC’s podoconiosis project since 2016.” (KII, IOCC staff) |
|  |  | Implementing actors | IOCC Ethiopia and NaPAN jointly implemented the project | The next Step project was implemented by IOCC and NaPPAN where IOCC was responsible for implementing the intervention and whereas NaPPAN was responsible for supervision and evaluation. “(KII, Regional NTD staff) |
|  |  | Intervention targets | The intervention targets individuals affected by podoconiosis, local community and health professionals. | - We had a plan to train health professionals and provide treatment for 7000 patients. We further provided health education at healthcare centers for the community (KII, staff of Intervention implementing organization) |
|  |  | Collaboration partnership and participation | Collaboration and commitment of government structures have been helpful implement the project.  Community participation is minimal. Bottom-up participation should be employed to maximize community mobilization and participation in the project. | - The Amhara Regional Health Bureau welcomed us when we inquired to recruit seconded staff and operate through integration approach along with the NTD team in the regional health office. As the NTD team is responsible to all types of the NTD related interventions, this approach created advantages in sharing resources and experience, minimizing resources duplications and sharing knowledges among the NTD team (KII, staff of intervention implementing organization). - Community participation was low. It would have been better if we could strongly work with the local influential people in the community to change attitude of the people”. (KII, NTD officer, Dera Woreda Health Office) |
|  | **Implementation outcomes**  Outcome is the result of the implementation effort. In this study, we focused on the fidelity, acceptability and sustainability of the intervention. Fidelity refers to what extent the implementation strategies are being implemented as per the plan. In addition, we will study to what extent the intervention is being accepted by the intended beneficiaries and what changes likely remain after the intervention. | Acceptability | **Knowledge about the purpose of the intervention**  Most of the patients in the community were well informed about the purpose of intervention and the types of services provided by the health center.  On contrary, the general community (most of the unaffected community members) seem to have limited information about the intervention or its purpose. | - Most patients have received education and attended the demonstration session at the health center...(KII, HEW Yilmana Densa Distric). - I have benefited from these interventions in a sense that my knowledge about the disease has been improved (IDI, affected, male age 54) - Patients hold better understanding than other people in the community…they have received information form the health center. I think healthy people have wrong understanding about it. (KII, Local Leader,Yilmana Densa district) |
|  |  |  | **Perception towards the significances of the intervention.**  Most respondents alluded intervention has introduced accessibility of treatment to podoconosis patients at the health centers. This initiative also created the impression among most patients and non-patients that podoconiosis disease can be treatable | In the past, we thought that this disease could not curable. Now that we have seen some indications that convinced us to believe the disease could be treatable and curable. Some patients have been recovered from it. (FGD participant, Women group, Yilmana Densa District) |
|  |  |  | **Demand creation and acceptance to treatment**  High enrollment /show up of patients and a considerable number graduating through the three months follow up period explains treatment acceptance among most patients. | - We did the mobilization door to door, at the church and in the schools to inform households to send podo patients to the clinic to receive the intervention. Accordingly, there was high turnout out of patients at the beginning.” (KII Podo focal staff ). - I went there three times, ones in every month last year. There were so many patients with me.” (IDI, young male patient, age 18.) |
|  |  | Fidelity.  Fidelity refers to what extent the implementation strategies are being implemented as per the plan. | As per the plan, the NGOs were able to train local health professionals and commenced the integration of podoconiosis within existing health facilities.  **Gaps:** Intervention was designed to cascade the treatment through the existing health structures. However, it mainly concentrated at the health center setting and not well trickled down to lower structure particularly in engaging Health Development army (HDA) and health extensions programs at the health posts | - We found that most of the health centers were doing well … Cascading trainings have been implemented in most of the health centers. In many of the health centers some patients show up regularly for follow up visits. High attendance means there is good linkage with the health extension workers. The review meeting informed us that there was a change in the reduction of acute attack among patients. (KII, staff of intervention implementing organization) - The intervention was not functional at the health post level. It was limited to the health center. We have limited follow up of patients after the three rounds of intervention at the health center (KII, NTD Officer, YD woreda Health Office). - We have not received any training on podoconiosis. It was given only to the health staff at the health center, not for health extension workers. We were given just a quick briefing or orientation by the health staff and observed the demonstrations performed by the health staff on how to wash and bandage the affected part of the feet. That was not sufficient for us to understand the condition in detail (KII, HEWs at Dera District) |
|  |  | Change on stigma: Reported Intervention impacts that likely contribute to changes on stigma. | Reduction of acute attack improved patients’ time to engage in economic activities. This resulted in turning around the prevailed sense of economic inferiority among patients. | - There is a stark difference compared to the past (before the intervention). A significant improvement has seen among many patients with regards to reduction in the magnitude of occurrence of acute attack and the offensive odor (KII, NTD Officer at Dera District Health Office). |
|  |  |  | The treatment has improved physical wellbeing among those adhering to the procedures in a sense that their footwear and feet hygiene practice boosted their self-worth and participation in social events and overcoming self-stigma. | - I have seen improvement among those who have received shoes and soap. Their feet became clean and neat. Unlike the past, their feet have no bad odor.” (IDI, affected male, age 40). |
|  |  |  | The intervention has introduced belief about treatability of podoconiosis. This has been noted by informants as important pathway to bring change on self-stigma of patients and public misconceptions. | - This intervention also sparked some light on patients understanding about the treatment procedures…also contributed for reduction of self-stigma and self-isolation of patients (KII-, NTD Officer, Yilmana Densa District) |
|  |  | Barriers to stigma reduction. | Prevailing misconceptions and lack of clear awareness about the cause of podoconiosis. (Stepping on goat’s blood, sharing washing tools, contacts with patients, etc.) | - Toilets harbor hook worms. Walking bare feet in these places put people at high risk for exposure to hookworms that eventually leads to podoconiosis” (KII, HEW, Dera District) - Hereditary swellings can’t be cured. It is the will of God (IDI, Affected male, age 40) - Mostly my clients report the main cause as washing the feet by the river side during hot seasons.” (KII, male traditional healer in District) |
|  |  |  | Lack of adherence to treatment: Expectation of instant cure from the treatment, high dependency syndrome and misunderstandings about the intervention accounted for many patients to drop out the treatment. The condition reinforced concerns over beliefs about curability of the disease that leverages the stigma reduction. | - I have given up as soon as the soap and ointments are run out...I cannot afford buying soaps leave alone these types of shoes. I am still waiting for shoe support” (IDI, Affected male age 64) |
|  |  |  | Economic challenge encountered by most patients influenced their access to treatment supplies and thwarted motivation to self-care. The economic burden also intensified inferiority feeling among patients manifested in social life. | - I didn’t hear about the intervention. I didn’t receive any supplies….[Why don’t you buy soap and wash your feet?]…Surely you jest ! Do you think I can buy soap every time to wash my feet?! |
|  |  | Sustainability | It was assumed that mainstreaming podoconiosis in the NTD structures and health centers reinforce sustainability of self-care treatment and ultimately improve the physical, social, and economic wellbeing of patients including stigma reduction.  It was assumed that patients will continue practicing the footcare instructions they were given during the intervention. Some patients lost trust on the treatment intervention due to lack of perseverance to the lengthy process required to see improvement. Such expectations to quick recovery have discouraged patients and to hold perception that podoconiosis is untreatable. | - The aim of the project was to ensure and strengthen integration of the podo operation in the target health centers” (KII, IOCC staff) - Integration was taken as a means for sustainability as it provides opportunity for creating access and open doors services for patients, which was also believed to eventually contribute to stigma reduction (KII, District NTD officer) - I tried to wash my feet and bandage it for a few weeks, it was useless… I thought it was curable, but I have not seen any change”. (IDI, Female patient, age 50) - The health professional told me that it is curable, but I don’t believe it is true. There is no promising improvement in my condition since I have started the treatments. I gave up hope all together. So, I don’t follow the instruction anymore (IDI, male, age 18) |

1. [↑](#footnote-ref-1)
2. [↑](#footnote-ref-2)
